# MIIP functions as a novel ligand for ITGB3 to inhibit angiogenesis and tumorigenesis of triple-negative breast cancer

**DOI:** 10.1038/s41419-022-05255-0

**Published:** 2022-09-21

**Authors:** Yujing Gao, Yujie Fang, Yongli Huang, Rui Ma, Xixi Chen, Fang Wang, Xiuying Pei, Yuanqi Gao, Xuehua Chen, Xinrui Liu, Jingxuan Shan, Pu Li

**Affiliations:** 1 National Health Commission Key Laboratory of Metabolic Cardiovascular Diseases Research, Ningxia Medical University, Yinchuan, China; 2 Key Laboratory of Fertility Preservation and Maintenance of Ministry of Education, Department of Biochemistry and Molecular Biology, School of Basic Medical Sciences, Ningxia Medical University, Yinchuan, China; 3 Ningxia Key Laboratory of Vascular Injury and Repair Research, Ningxia Medical University, Yinchuan, China; 4 Department of Pediatrics, Ruijin Hospital, Shanghai Jiao Tong University School of Medicine, Shanghai, China; 5 Department of Gastroenterology, General Hospital of Ningxia Medical University, Yinchuan, China; 6 Department of Genetic Medicine, Weill Cornell Medicine, New York, NY USA

**Keywords:** Tumour-suppressor proteins, Breast cancer

## Abstract

Migration and invasion inhibitory protein (MIIP) has been identified as a tumor suppressor in various cancer types. Although MIIP is reported to exert tumor suppressive functions by repressing proliferation and metastasis of cancer cells, the detailed mechanism is poorly understood. In the present study, we found MIIP is a favorable indicator of prognosis in triple-negative breast cancer. MIIP could inhibit tumor angiogenesis, proliferation, and metastasis of triple-negative breast cancer cells in vivo and in vitro. Mechanistically, MIIP directly interacted with ITGB3 and suppressed its downstream signaling. As a result, β-catenin was reduced due to elevated ubiquitin-mediated degradation, leading to downregulated VEGFA production and epithelial mesenchymal transition. More importantly, we found RGD motif is essential for MIIP binding with ITGB3 and executing efficient tumor-suppressing effect. Our findings unravel a novel mechanism by which MIIP suppresses tumorigenesis in triple-negative breast cancer, and MIIP is thus a promising molecular biomarker or therapeutic target for the disease.

## Introduction

Breast cancer is the most commonly diagnosed cancer and the leading cause of death by cancer among females worldwide, with an estimated 2.3 million cases and 685,000 deaths in 2020 [1]. The diagnosis and treatment of breast cancer has made great progress in recent years. However, the development of drug resistance after long-term medication limits the sustainability of therapeutic efficacy. Worse still, for triple-negative breast cancer (TNBC), due to lack of specific therapeutic targets, the prognosis is poorer than other subtypes of breast cancer. Therefore, it is necessary to reveal the mechanism of the occurrence and development of breast cancer, so as to discover and identify novel biomarkers or therapeutic targets for early diagnosis and treatment of the disease.

MIIP was firstly identified as an IGFBP2-interacting protein [2]. The gene encoding MIIP locates on chromosome 1p36.22, a locus commonly deleted in many tumors [3]. So far, many studies reported the anti-tumor function of MIIP in different cancer types [3–9]. Meanwhile, MIIP might also have tumor-promoting ability, evident by the findings that MIIP was highly expressed in the esophageal squamous cell carcinoma tissues compared to adjacent normal tissues [10]; and phosphorylation of MIIP at Ser303 facilitated metastasis of colorectal cancer [11]. Therefore, MIIP might play distinct roles in a context-dependent manner. The reported mechanism for the tumor-suppressive role of MIIP includes reducing the stability and activity of HDAC6 [12], interacting with CDC20 to interfere with the function of APC/C acting as an ubiquitin-ligase E3 for cyclin B1 degradation [13], interaction with PP1α and negative modulating AKT signaling [14], and regulating activity and expression of NF-κB [2, 11]. However, it is not fully elucidated yet.

Integrins are multifunctional heterodimeric cell-surface receptor molecules linking cells to counter-receptors on other cells and ligands in the extracellular matrix (ECM) [15, 16]. The β3 integrin (ITGB3) has been reported to play critical roles in tumorigenesis by reprogramming tumor metabolism, promoting angiogenesis, facilitating epithelial to mesenchymal transition (EMT), and maintaining tumor stemness [15, 17]. ITGB3 has two forms of heterodimer, platelet integrin αIIbβ3 and endothelial integrin αVβ3, both of which recognizes ligands containing the RGD tripeptide motif, such as vitronectin and fibronectin [18, 19]. Integrin αVβ3 overexpression was observed in angiogenetic endothelial cells [20] and tumor cells. In highly metastatic melanoma, high expression of integrin αVβ3 is closely associated with malignant phenotype [21]. Binding of integrin αVβ3 with vitronectin promotes proliferation, adhesion and motility of ovarian cancer cells [22]. Hence, integrin αVβ3 is regarded as a promising therapeutic target for cancer treatment, with the development of Cilengitide (a cyclic RGD-f-(NMe)V peptide), Etaracizumab (a mAb specific against αVβ3), MK-0429, and other ITGB3 inhibitors, some of which have shown encouraging outcomes in phase I/II clinical trials [23–25].

In the present study, we demonstrated MIIP inhibits tumorigenesis of TNBC by suppressing tumor angiogenesis, as well as cell proliferation and migration. Mechanistically, MIIP suppresses activation of ITGB3 downstream signaling by directly interacting with ITGB3 and promoting its degradation, leading to decreased phosphorylation of FAK and AKT. As a result, β-catenin is reduced due to increased ubiquitin-mediated degradation, which contributes to downregulated VEGFA production and EMT transformation. More importantly, we found RGD motif is essential for the efficient tumor-suppressing capacity of MIIP. These findings not only unravel a novel mechanism by which MIIP suppresses tumorigenesis in TNBC, but also provide MIIP as a promising molecular biomarker or therapeutic target for the disease.

## Results

### MIIP is a favorable prognostic indicator for TNBC

**Fig. 1:**
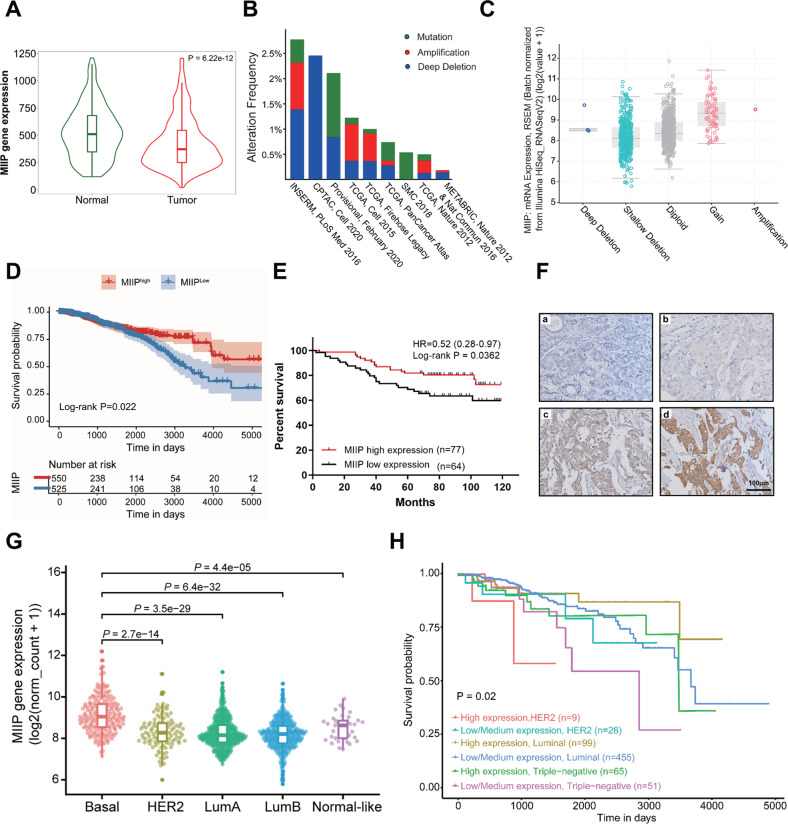
MIIP is a favorable prognosis indicator in patients with TNBC. **A** Comparison of MIIP expression between normal tissues (Normal) and breast cancer tissues (Tumor) was performed in the TNMplot database (https://tnmplot.com/analysis/). **B** MIIP gene alteration (amplification (red), deep deletion (blue), and mutation (green)) across different breast cancer cohorts in the TCGA database. **C** Relationship between MIIP mRNA expression levels and DNA copy-number alterations in TCGA breast cancers. **D** Kaplan-Meier survival analysis for breast cancer patients with high- or low- MIIP expression levels using data retrieved from TCGA database (*n* = 1075). **E** Kaplan-Meier analysis for the overall survival of 141 breast cancer patients with a median age of 58 years (range 33–88 years), stratified according to MIIP protein level (high or low). **F** Representative immunohistochemical staining of MIIP in a breast cancer tissue array. **a** negative staining; **b**, weak positive staining of MIIP; **c**, moderate positive staining of MIIP; **d** strong positive staining of MIIP. **G** MIIP expression across different PAM50 subtypes of breast cancer was compared using data retrieved from TCGA database. Basal, basal-like; HER2, HER2-enriched, LumA, Luminal A; LumB, Luminal B. **H** Effect of MIIP expression level & cancer type on breast cancer (BRCA) patient survival was analyzed using UALCAN Database.

To investigate the role of MIIP in breast cancer, we first analyzed the expression and copy number alteration of MIIP in breast cancer using public databases. As shown in Fig. 1A, expression of MIIP in breast cancer is lower than in normal tissues. Among the different MIIP genomic alterations, gene deletion and mutation are the relatively frequent events in invasive breast cancer (Fig. 1B), and the MIIP gene locus has relatively high frequency of copy number loss (Fig. S1). Additionally, MIIP shallow deletion is correlated with decreased MIIP mRNA expression, which occurs in majority of breast cancer samples (Fig. 1C). The results from the survival analysis using TCGA database and immunohistochemistry staining of a breast cancer tissue array suggested that patients with high expression of MIIP had a better survival outcome (Fig. 1D–F). When analyzing MIIP expression among different prediction analysis of microarray 50 (PAM50) subtype tumors, we found basal-like tumors had relatively higher expression of MIIP (Fig. 1G), implying the crucial role of MIIP in this subtype of breast cancer. We then analyzed the relationship between MIIP expression and the survival probability of patients with different subtype breast cancer using the UALCAN database, which showed that patients with high expression of MIIP still had a better survival probability than those with low MIIP expression in TNBC (Fig. 1H), the most malignant subtype of breast cancer, suggesting MIIP also functions as a tumor suppressor in TNBC.

### MIIP inhibits tumorigenesis of TNBC cells in vitro and in vivo

**Fig. 2:**
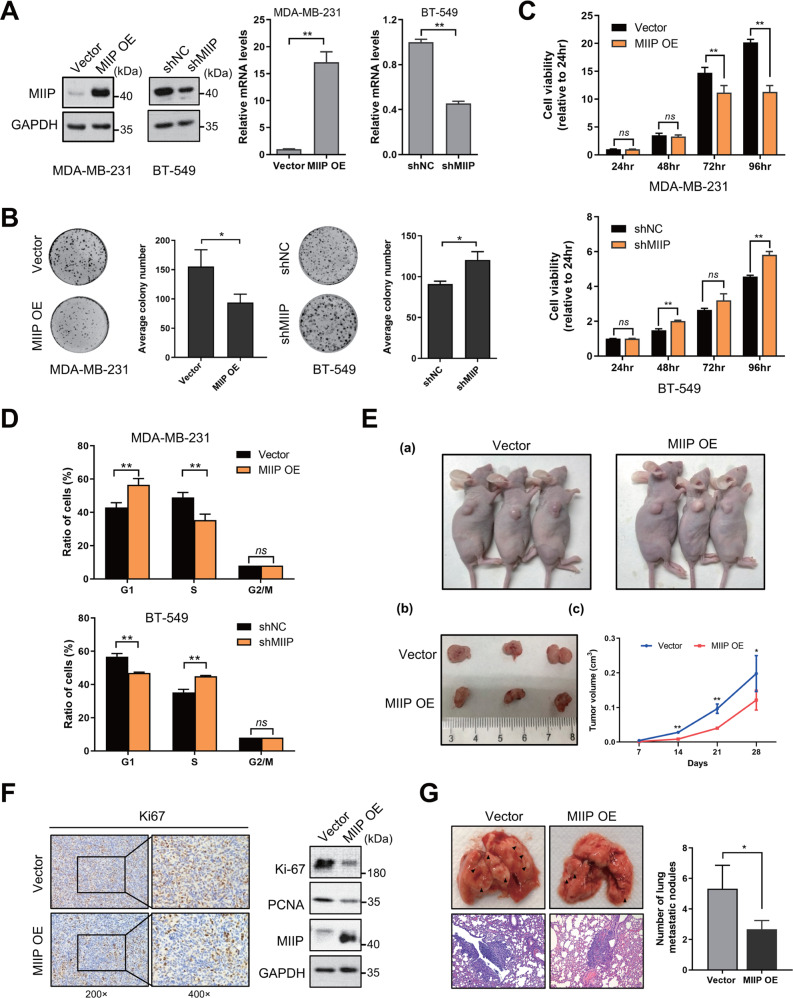
MIIP inhibits tumorigenesis of TNBC cells. **A** Expression levels of MIIP were examined by western blot and QPCR in MDA-MB-231 cells or BT-549 cells after MIIP expression was overexpressed or knocked down. **B** Colony formation assay was performed in MDA-MB-231 cells and BT-549 cells after MIIP expression was altered. **C** Cell viability was tested by MTS assay to determine the growth of MDA-MB-231 and BT-549 cells after MIIP expression was altered. **D** Cell cycle of MDA-MB-231 and BT-549 cells with altered MIIP expression was analyzed by flow cytometry. Ratios of cells in each cell phase were obtained using ModFit LT 3.0. **E** MDA-MB-231 cells with or without MIIP overexpression were injected subcutaneously into nude mice. Representative images of the tumor-bearing mice and tumor masses are shown. Tumor size was monitored regularly. **F** Protein levels of MIIP, Ki67, and PCNA in the tumor tissues from **E** were examined by western blot or immunohistochemistry. **G** MDA-MB-231 cells with or without MIIP overexpression were subjected to mouse xenograft through tail vein injection. Lung metastases were observed and calculated; arrows indicate examples of metastatic nodules. Data are presented as mean values ± SD. *: *P* < 0.05; **: *P* < 0.01.

To determine the role of MIIP in TNBC, firstly, we stably overexpressed MIIP or knockdown of MIIP in TNBC cells MDA-MB-231 or BT-549 (Fig. 2A). The colony formation assay result showed that the number and size of cell colonies was noticeably reduced when MIIP was overexpressed, while knockdown of MIIP displayed opposite effect (Fig. 2B). Meanwhile, overexpression of MIIP significantly inhibited the growth of MDA-MB-231 cells, especially at 72 hr and 96 hr time points, while MIIP knockdown promoted growth of BT-549 cells (Fig. 2C). In addition, ectopic MIIP expression in MDA-MB-231 cells led to an obvious G1 phase arrest of cell cycle. In contrast, after knockdown of MIIP in BT549 cells, the ratio of cells in G1 phase was significantly decreased, while that in S phase was increased (Fig. 2D, Fig. S2A). The above results indicate MIIP could inhibit growth of TNBC cells in vitro. We pursued this effect further by a xenograft model. Consistently, MDA-MB-231 cells with MIIP overexpression grew slower and formed smaller tumor masses than the empty vector-transfected control cells in nude mice (Fig. 2E), and tumor tissues formed from MIIP-overexpressing cells had a lower expression of Ki67 and PCNA (Fig. 2F); whereas knockdown of MIIP accelerated in vivo growth of BT549 cells (Fig. S2B). Furthermore, using a lung metastasis mouse model, we observed MIIP overexpression could substantially inhibit metastasis of MDA-MB-231 cells in the lungs of the mice (Fig. 2G). Collectively, these results demonstrate MIIP suppresses tumorigenesis of TNBC cells in vitro and in vivo.

### MIIP inhibits tumor angiogenesis and cell migration of TNBC

**Fig. 3:**
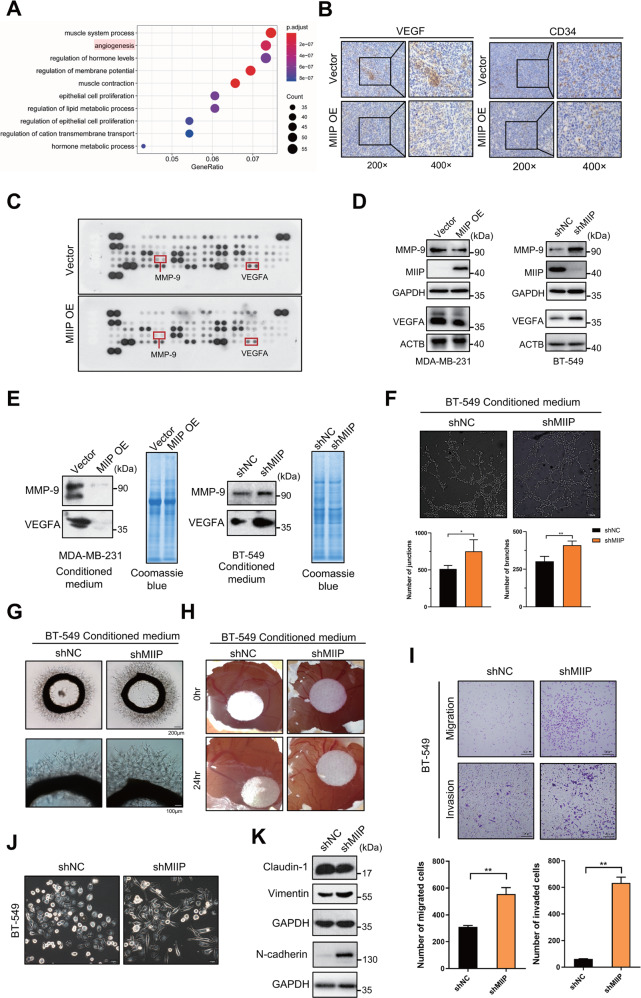
MIIP inhibits tumor angiogenesis and cell migration of TNBC. **A** Gene Ontology analysis of downregulated genes in MIIP^high^ expression group relative to MIIP^low^ expression group using data retrieved from TCGA database. **B** Expression of CD34 and VEGF in the tumors formed from MDA-MB-231 cell with MIIP overexpression or the control cells were determined by immunohistochemistry. **C** Human angiogenesis array was performed to determine changes of angiogenesis-related factors in MDA-MB-231 cells after MIIP was overexpressed. **D** Effect of MIIP on the expression of VEGFA and MMP-9 was validated by western blot in MDA-MB-231 cells and BT-549 cells. **E** Levels of VEGFA and MMP-9 in the conditioned medium (with same amount in total protein) of MDA-MB-231 cells and BT-549 cells with altered MIIP expression were determined by western blot. Coomassie blue staining of the gel was applied to serve as loading control. **F** Tube formation assay of HUVEC cells treated with conditioned medium (4 μg in protein amount) from BT-549 cells with altered MIIP expression. Effect of conditioned medium (4 μg in protein amount) from BT-549 cells with or without MIIP knockdown on the ex vivo angiogenesis was evaluated by **G** rat aortic ring assay and **H** chick embryo chorioallantoic. **I** Transwell assay was performed to investigate the abilities of migration and invasion in BT549 cells with indicated stable transfection. **J** Morphology of BT-549 cells with altered MIIP expression was observed under the bright field of inverted microscope. **K** Expression levels of EMT-related proteins in BT-549 cells stably transfected with shRNA targeting MIIP (shMIIP) or negative control shRNA (shNC) were determined by western blot. Data are presented as mean values ± SD. *: *P* < 0.05; **: *P* < 0.01.

To identify the possible mechanism by which MIIP inhibits tumorigenesis in TNBC, we analyzed the pathways associated with MIIP by Gene Ontology analysis using data retrieved from TCGA. As shown in Fig. 3A, pathways including muscle system process, regulation of hormone levels, epithelial cell proliferation and lipid metabolic process were related to MIIP; particularly, angiogenesis was one of the most obvious pathways associated with MIIP. Given angiogenesis is a fundamental and crucial event for tumor growth, which has been characterized as one of the hallmarks of cancer [26] and the promising target for cancer therapy, we wondered whether MIIP inhibits growth of TNBC via suppressing tumor angiogenesis. To this end, we first detect expression of two classic markers for angiogenesis, CD34 and VEGF, in tumor tissues formed from MDA-MB-231 cells with or without MIIP overexpression. As shown in Fig. 3B, tumor tissues formed from MIIP-overexpressing MDA-MB-231 cells had decreased levels of CD34 and VEGFA, compared with that from control cells. Next, human angiogenesis array was performed to determine effects of MIIP on the expression of angiogenesis-related factors. As shown in Fig. 3C and Fig. S3A, overexpression of MIIP could markedly reduce protein levels of many angiogenesis-related factors. We then selected VEGFA and MMP-9 for further validation, owing to their critical roles in angiogenesis. Consistently, MIIP overexpression could definitely decrease VEGFA and MMP-9 protein levels, whereas knockdown of MIIP elevated MMP-9 and VEGFA levels (Fig. 3D).

Moreover, we collected the conditioned medium (CM) of TNBC cells (Fig. S3B), and tested the levels of VEGFA and MMP-9 in the CM, which also showed decreased levels of VEGFA and MMP-9 in the CM of MIIP-overexpressing MDA-MB-231 cells; whereas VEGFA and MMP-9 levels were higher in the CM from BT-549 cells with MIIP knockdown than that from control cells (Fig. 3E). Next, we investigated the effect of these CM on angiogenesis. The results showed that in comparison with CM from control cells, those from MIIP-knockdown BT-549 cells obviously promoted tube formation of HUVECs (Fig. 3F), outgrowth of microvessels from the rat aortic rings (Fig. 3G) and vascularization of the chick embryo chorioallantoic membrane (Fig. 3H).

In addition, we confirmed that knockdown of MIIP could promote migration and invasion abilities of TNBC cells using wound healing assay and transwell chamber assay (Fig. S3C, Fig. 3I), which is attributed to promotion of EMT by MIIP knockdown, as evidenced by the spindle-like and fibroblastic morphology of BT-549 cells with MIIP knockdown, and the expression patterns of EMT-related markers (decreased expression of claudin-1, increased expression of N-cadherin and Vimentin) in BT-549 cells with MIIP knockdown (Fig. 3J, K).

Collectively, the aforementioned results indicate MIIP inhibits tumor angiogenesis and migration via downregulation of pro-angiogenesis factors and EMT in TNBC.

### ITGB3/β-catenin signaling is suppressed by MIIP in TNBC

**Fig. 4:**
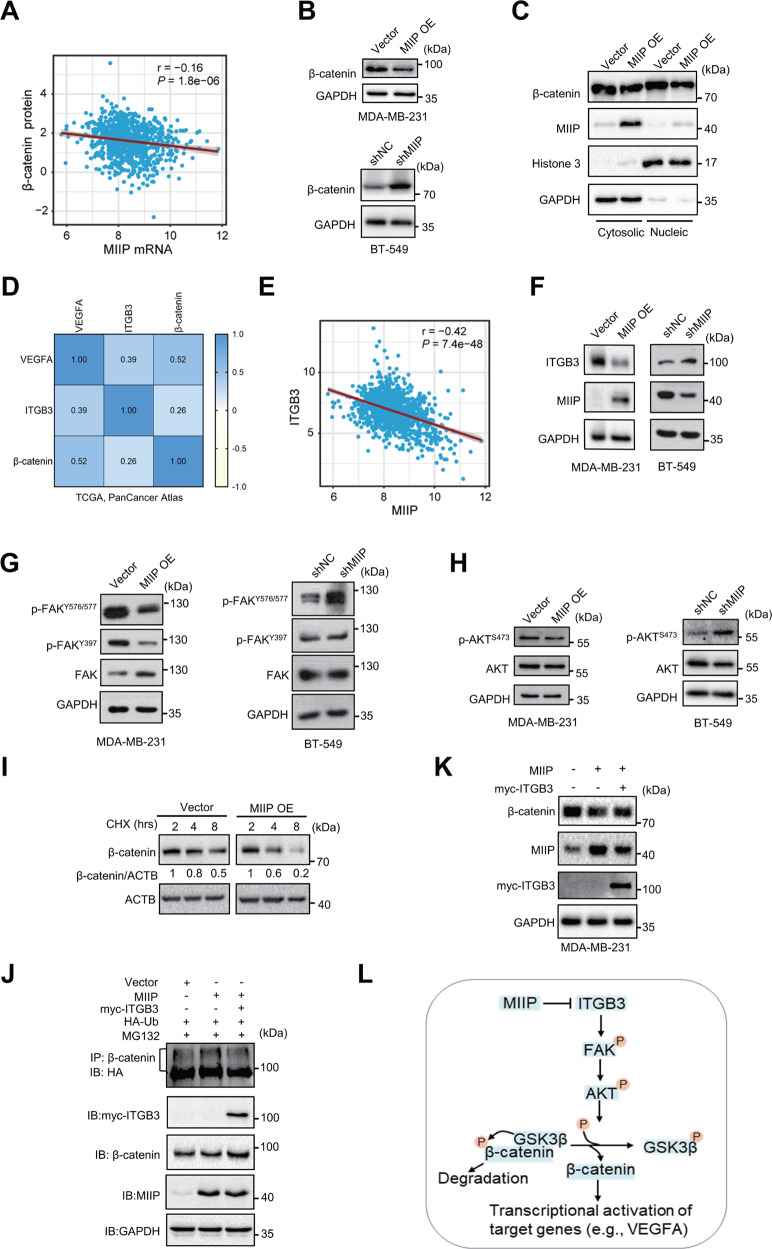
MIIP suppresses ITGB3/β-catenin signaling in TNBC. **A** Correlation of MIIP mRNA expression and β-catenin protein was analyzed using data retrieved from TCGA database. **B** Protein levels of β-catenin were investigated by western blot in TNBC cells with altered MIIP expression. **C** Effect of MIIP on cytosolic and nucleic protein levels of β-catenin was determined by western blot. **D** Reciprocal correlation of the protein levels of VEGFA, ITGB3 and β-catenin was analyzed using cBioPortal breast invasive carcinoma dataset (TCGA, PanCancer Atlas). **E** Correlation of MIIP expression and ITGB3 expression in breast cancer tissues was analyzed using data retrieved from TCGA database. **F** Impact of MIIP on ITGB3 expression in TNBC cells was determined by western blot. **G** Activity of the ITGB3 downstream FAK signaling in TNBC cells with altered MIIP expression was evaluated by western blot. **H** Activity of AKT in TNBC cells with altered MIIP expression was investigated by western blot. **I** Stability of β-catenin in MDA-MB-231 cells with or without MIIP overexpression under indicated 100 μM CHX (cycloheximide, the protein synthesis inhibitor) treatment was analyzed by western blot. **J** Ubiquitination level of β-catenin was analyzed in MDA-MB-231 cells co-transfected with indicated vectors. **K** β-catenin protein level was determined by western blot in MDA-MB-231 cells co-transfected with MIIP-expressing vectors and ITGB3-expressing vectors or empty vectors. **L** A schematic presentation showing MIIP suppresses ITGB3/FAK/AKT/β-catenin signaling.

Considering β-catenin could transcriptionally activated VEGFA and MMPs to regulate tumor angiogenesis and metastasis, we wondered whether β-catenin is implicated in MIIP-elicited tumor-suppressing effect in TNBC. Through analysis of the TCGA breast cancer database, we identified a negative correlation between MIIP mRNA levels and β-catenin protein levels in breast cancer samples (Fig. 4A). The negative regulation of β-catenin by MIIP was validated in TNBC cells (Fig. 4B). As a transcription factor, β-catenin could translocate from cytoplasm to nucleus to regulate expression of target genes, including VEGFA and MMP-9. We then detected the distribution of β-catenin in cytoplasm and nucleus, which showed that both cytosolic and nucleic β-catenin levels were reduced by MIIP (Fig. 4C).

ITGB3 has been reported to play an important role in promoting tumor angiogenesis through enhancing transcriptional activation of VEGFA by β-catenin. Using the TCGA PanCancer Atlas data, we found a positive correlation between the protein levels of ITGB3, β-catenin and VEGFA **(**Fig. 4D**)**. Simultaneously, MIIP was negatively correlated with ITGB3 in expression in breast cancer (Fig. 4E). Consistently, protein levels of ITGB3 was decreased by MIIP in TNBC cells (Fig. 4F). Moreover, we tested the effect of MIIP on the activity of FAK, the classic downstream signal molecule of ITGB3, which showed that MIIP overexpression suppressed the activation of FAK, as evidenced by the reduced phosphorylation levels of FAK at Y576/577 and Y397; whereas knockdown of MIIP had an opposite effect (Fig. 4G). Activation of FAK is capable to activate AKT, which further phosphorylates GSK3β and prevents it from promoting phosphorylation and ubiquitin-mediated degradation of β-catenin. We therefore investigated whether MIIP could further affect AKT activation and β-catenin degradation. As shown in Fig. 4H, MIIP decreased phosphorylation of AKT at S473; simultaneously, protein stability of β-catenin was reduced in MIIP-overexpressing MDA-MB-231 cells compared with control cells (Fig. 4I). More importantly, recovery of ITGB3 expression in MIIP-overexpressing cells could partially attenuate enhanced ubiquitination and decreased protein levels of β-catenin by MIIP (Fig. 4J, K).

Taken together, the aforementioned results indicate MIIP could induce β-catenin degradation through suppressing ITGB3 signaling, which further inhibits β-catenin-dependent transcriptional activation of target genes (Fig. 4L).

### MIIP directly interacts with ITGB3 to inhibit its downstream signaling

**Fig. 5:**
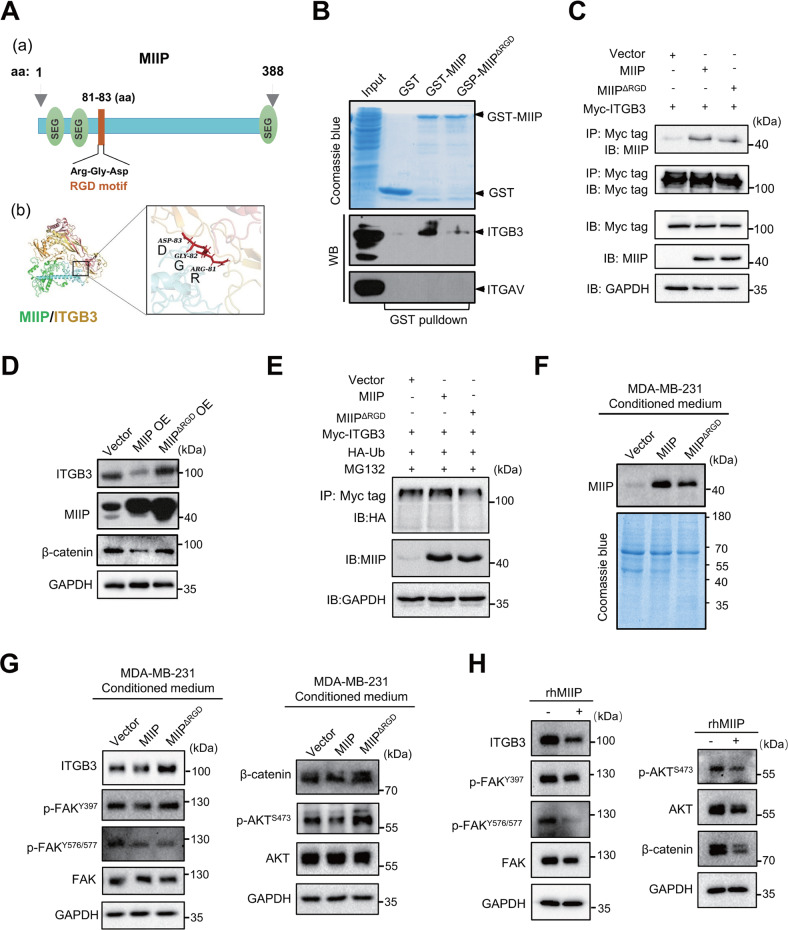
MIIP interacts with ITGB3 to suppress its downstream signaling. **A** A schematic presentation of MIIP protein structure **a**; **b** Computational modeling illustrating binding between MIIP and ITGB3; RGD motif of MIIP is visualized as a stick. **B** GST pulldown was performed to determine the direct interaction between ITGB3 or ITGAV and MIIP or MIIP with deleted RGD motif (MIIP^△RGD^). **C** Interaction between MIIP or MIIP^△RGD^ mutant and ITGB3 was investigated by Co-IP in 293 T cells. **D** Protein levels of ITGB3 and β-catenin were measured by western blot in MDA-MB-231 cells with MIIP or MIIP^△RGD^ overexpression. **E** Ubiquitination level of ITGB3 was analyzed in MDA-MB-231 transfected with indicated vectors. **F** Protein levels of MIIP in the conditioned medium of MDA-MB-231 cells stably transfected with indicated constructs were determined by western blot. Coomassie blue staining of the gel was applied to serve as loading control. **G** Effect of the conditioned medium from MDA-MB-231 cells on ITGB3/FAK/AKT/β-catenin signaling was investigated by western blot. **H** Effect of the human recombinant MIIP protein (rhMIIP) on ITGB3/FAK/AKT/β-catenin signaling in MDA-MB-231 cells was investigated by western blot.

ITGB3 could directly interact with proteins containing RGD (Arg-Gly-Asp) motif, such as fibronectin and vitronectin [15], which further play essential roles in tumor angiogenesis. Many compounds were designed based on their interaction to inhibit angiogenesis for cancer therapy [27]. We noticed a RGD motif (Arg81-Gly82-Asp83) existed within the structure of MIIP (Fig. 5A), prompting us to investigate whether MIIP directly interacts with ITGB3. To address it, we initially analyzed the potential interaction possibility between MIIP and ITGB3 using a docking model generated by Zdock program, which showed MIIP is in complex with ITGB3 (PDB ID: 6BXJ) at Arg81, Gly82, and Asp83, with an E_Rdock score of 3.0104 (Fig. 5A, Fig. S4A). Then, GST-pulldown was performed for further validation. In line with the result of docking model, GST-MIIP could pulldown ITGB3, but not ITGAV, the predominant counterpart of ITGB3 in tumor cells, indicating the direct interaction between MIIP and ITGB3 (Fig. 5B), which is consistent with the reports that β3 integrin contains the RGD ligand-binding site and RGD motif binds primarily to the β subunit of ITGAvB3 integrin [25, 28]. In addition, ITGB3 could co-purified MIIP (Fig. 5C). Of note, this interaction was markedly decreased when RGD motif of MIIP was deleted (Fig. 5B, C). Moreover, deletion of RGD motif noticeably disrupted the suppressive function of MIIP in regulating ITGB3 and β-catenin expression (Fig. 5D). Interestingly, we found proteasome inhibitor MG132 could partially reverse MIIP-induced reduction of ITGB3 protein level (Fig. S4B); in addition, MIIP enhanced ubiquitination of ITGB3, while RGD deletion partially attenuated this effect (Fig. 5E), implying MIIP interacts with ITGB3 via RGD motif to promote ubiquitination and degradation of ITGB3, and thereby suppress downstream β-catenin activity.

Given many of the RGD motif-containing ligands for ITGB3 are components of extracellular matrix, we wondered whether MIIP could be secreted out of the cells and inhibits ITGB3 signaling in an autocrine/paracrine manner. To illustrate it, firstly, we measured the existence of MIIP in CM of TNBC cells. As shown in Fig. 5F and Fig. S4C, MIIP could be detected in the CM of the cells, indicating secretion of MIIP by the cells. Moreover, we assessed the effects of the CM from MDA-MB-231 cells with overexpression of wild type MIIP or RGD motif-deleted MIIP on the activity of ITGB3 signaling. The result showed an inhibition of ITGB3 signaling by the CM of MIIP-overexpressing cells, while this effect was diminished when RGD motif of MIIP was deleted (Fig. 5G). For further validation, we tested the effects of recombinant human MIIP proteins (rhMIIP) in MDA-MB-231 cells. As shown in Fig. 5H, treatment of rhMIIP indeed inhibited ITGB3 signaling, as evidenced by decreased phosphorylation of FAK and AKT. In addition, ITGB3 protein level was also decreased by rhMIIP treatment (Fig. 5H). By using the protein synthesis inhibitor cycloheximide (CHX) or proteasome inhibitor MG132, we further corroborated rhMIIP elevated degradation and ubiquitination of ITGB3 (Fig. S4D, E), which is in line with the reports that ligands binding and FAK activation could regulate the ubiquitination and turnover of integrin [16, 29, 30].

Taken together, these results indicate MIIP could be secreted out of TNBC cells and bind with ITGB3 to promote its degradation and suppress its downstream signaling.

### Deletion of RGD motif impairs tumor-suppressing effect of MIIP in TNBC

**Fig. 6:**
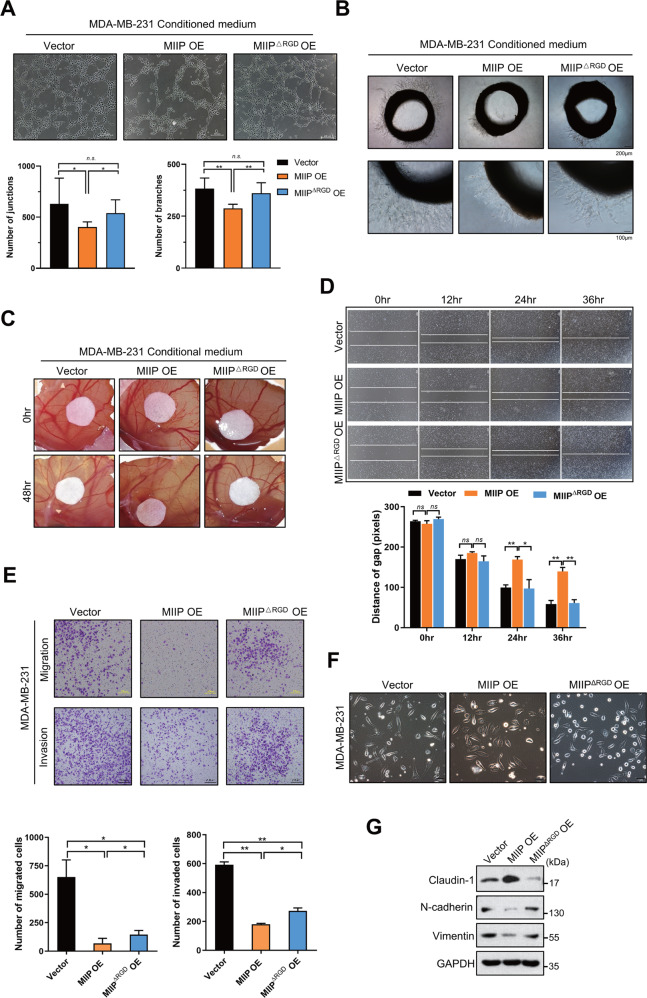
RGD motif is essential for MIIP to execute suppressive effect on angiogenesis and cell migration of TNBC. **A** Tube formation assay of HUVEC cells treated with conditioned medium from MDA-MB-231 cells with MIIP or MIIP^△RGD^ overexpression (4 μg in protein amount). **B** Rat aortic ring assay and **C** Chick embryo chorioallantoic assay were performed to evaluate the effect of the conditioned medium from MDA-MB-231 cells with MIIP or MIIP^△RGD^ mutant overexpression on the ex vivo angiogenesis. **D** Wound healing assay was performed to evaluate the migration ability of MDA-MB-231 cells with indicated stable transfection. **E** Transwell assay was performed to investigate the abilities of migration and invasion in MDA-MB-231 cells with indicated stable transfection. **F** Morphology of MDA-MB-231 cells stably transfected with MIIP-expressing, MIIP^△RGD^-expressing or empty vectors was observed under the bright field of inverted microscope. **G** Expression levels of EMT-related proteins in MDA-MB-231 cells stably transfected with MIIP-expressing, MIIP^△RGD^-expressing or empty vectors were determined by western blot. Data are presented as mean values ± SD. *: *P* < 0.05; **: *P* < 0.01.

Next, we investigated the necessity of RGD motif for the angiogenesis-suppressive effect of MIIP. As shown in Fig. 6A–C, MIIP with deleted RGD motif diminished the ability to inhibit tumor angiogenesis, as evidenced by the results from HUVECs tube formation assay, chick embryo chorioallantoic membrane assay, and rat aortic ring assay. Consistently, migration and invasion ability of MDA-MB-231 cells with MIIP overexpression was lower than that of control cells, whereas deletion of RGD motif compromised these inhibitory effects of MIIP on migration and invasion in TNBC cells (Fig. 6D, E). Moreover, we detected changes in cell morphology and levels of molecules associated with EMT after MDA-MB-231 cells was transfected with MIIP- or MIIP ^ΔRGD^-expressing vectors. The results indicated that overexpression of MIIP but not MIIP with RGD motif deletion could change the morphology of MDA-MB-231 cells to a more cobblestone-like appearance (Fig. 6F); and expression of the epithelial marker claudin-1 was increased, while that of the mesenchymal markers (N-cadherin and Vimentin) were decreased by MIIP, but not MIIP with deleted RGD motif (Fig. 6G).

Collectively, the above results suggest RGD motif is critical for MIIP to suppress tumorigenesis of TNBC.

## Discussion

MIIP was characterized by its role in inhibiting migration and invasion in glioma, and hence the name. Since the identification of MIIP [2], a limited number of studies addressed the function and mechanism of MIIP in tumorigenesis, with most of them indicate MIIP plays inhibitory effects on tumorigenesis in many types of cancer [6–9, 14, 31–34]. Besides, a few study also reported opposite effects of MIIP. For instance, positive correlation of MIIP high expression with poor prognosis was observed in esophageal squamous cell carcinomas [10]; and phosphorylation of MIIP at Ser303 by PKCε could enhance RelA transcriptional activity and thus promote metastasis of colorectal cancer [11]. In breast cancer, the case-control study for the SNPs or LOH of *MIIP*, and the association between MIIP expression and clinical prognosis factors or cell biologic behaviors suggested the involvement of MIIP in breast cancer development and progression [4, 5, 35]. However, the exact role of MIIP in breast cancer or even in cancer is not well understood yet. In our current study, we confirmed MIIP inhibits in vitro and in vivo growth of TNBC cells, as well as cell migration and invasion, which is consistent with the conclusions obtained from previous studies that MIIP functions as a tumor suppressor. Interestingly, MIIP expression was higher in TNBC than in other subtypes of breast cancer. One possible explanation for this phenomenon is that MIIP expression is passively enhanced during the tumorigenesis in a TNBC specific way in order to counteract the development of the disease, thus resulting in a relatively high basic expression level of MIIP in TNBC. Future in-depth studies are needed to clarify the detailed mechanism.

As one of the characteristics of cancer cells [26], angiogenesis is the biological basis and crucial element for malignant transformation, tumor growth and metastasis [36]. VEGF/VEGFR pathway is the major signaling that promotes tumor angiogenesis, especially VEGFA and its ligand VEGFR2, which have definite and critical function in tumor angiogenesis and have been utilized as therapeutic targets for cancer treatment [37, 38]. Here, we reported for the first time that MIIP represses tumor angiogenesis by inhibiting production of VEGFA in TNBC cells. Previously, we also found overexpression of MIIP in HUVECs could suppress tube formation and cell migration [39]. Therefore, we provide a novel understanding on the function of MIIP during tumorigenesis in the aspect of tumor angiogenesis.

The existed RGD motif within MIIP protein structure promoted us to investigate the relationship between MIIP and ITGB3, the essential component of RGD receptors. We confirmed a direct interaction between MIIP and ITGB3. Moreover, overexpression of MIIP suppressed activation of FAK, the downstream molecule of ITGB3. Integrins are multifunctional heterodimeric cell-surface receptor molecules that mediates adhesion between cells or cells and extracellular matrix (ECM). More than twenty integrins can be assembled from distinct α subunits and β subunits, serving as receptors to recognize and bind different ECM proteins and initiate intracellular signaling pathways, which eventually elicit biological effects like cell adhesion, invasion and migration. The ITGB3 interacts with integrin αIIb or αV forming integrin αIIbβ3 or αVβ3, with αIIbβ3 predominantly expressed in platelet while αVβ3 predominantly expressed in endothelial cells or tumor cells. Due to the important role of ITGB3 in angiogenesis, it has been the target for anti-angiogenesis therapy, such as Cilengitide and the mAb specific against αVβ3, Etaracizumab. Here, we demonstrated MIIP is an endogenous inhibitor for ITGB3, which at least partially contributes to the inhibitory effect of MIIP on angiogenesis. In addition, interaction of ITGB3 and MIIP is dependent on the RGD motif of MIIP, and MIIP with deleted RGD motif diminishes its antiangiogenic ability.

As a transcription factor, β-catenin could activate the transcription of several tumor-promoting molecules, including VEGFA, MMPs and EMT-related proteins [40–42], which contribute to enhanced tumor metastasis capacity [43]. The stability of β-catenin is governed by GSK3β, whose activity is regulated by AKT activation. ITGB3 could affect the stability of β-catenin by its downstream FAK/AKT signaling. Here, we found MIIP expression level is negatively correlated with the protein level of β-catenin in TNBC, and overexpression of MIIP could enhance degradation of β-catenin. Moreover, ITGB3 overexpression could partially attenuate downregulation of β-catenin by MIIP; and MIIP with deleted RGD motif lost the inhibitory effect on EMT and angiogenesis. Therefore, ITGB3/AKT/β-catenin axis is downregulated by MIIP to suppress tumor angiogenesis and EMT.

More importantly, our data for the first time indicated the existence of MIIP in the CM of tumor cells, indicating MIIP could be secreted by the cells and function as a ligand of ITGB3. Furthermore, we confirmed that both the CM of TNBC cells and the recombinant human MIIP protein could elicit changes in ITGB3 downstream signaling. However, further study is needed to systematically validate the ligand-receptor relationship between MIIP and ITGB3.

**Fig. 7:**
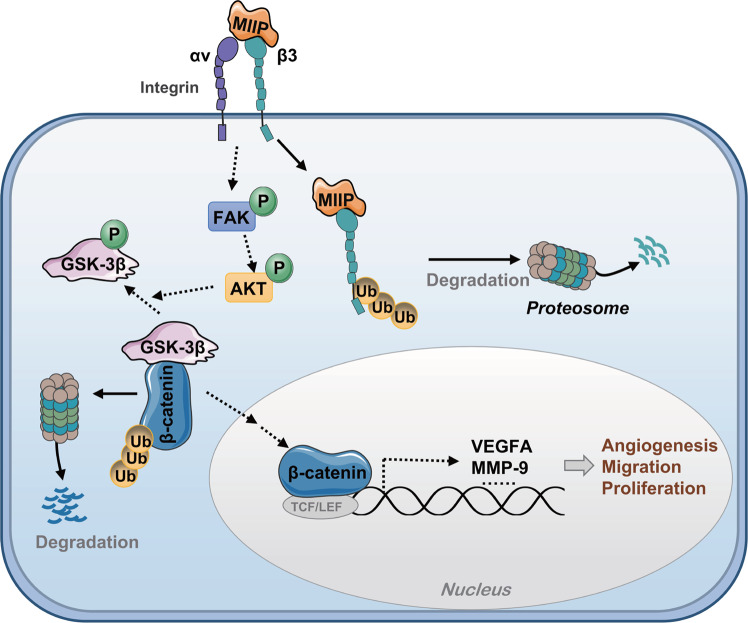
The mechanistic diagram of MIIP function in TNBC. Proposed model of MIIP inhibiting tumorigenesis of TNBC through suppressing ITGB3/β-catenin signaling.

In conclusion, our study demonstrates that MIIP could inhibit angiogenesis, proliferation and migration of TNBC cells in vitro and in vivo. MIIP suppresses activation of ITGB3 signaling by directly binding with ITGB3 and promoting its degradation. Degradation of β-catenin is a critical downstream event of MIIP/ITGB3 to suppress the expression of VEGFA and EMT-related proteins (Fig. 7). Our findings not only reveal a novel mechanism by which MIIP suppresses tumorigenesis in TNBC, but also provide MIIP as a promising molecular biomarker or therapeutic target for the disease.

## Materials and methods

### Plasmids and recombinant MIIP protein

shRNA targeting MIIP (shMIIP) and a negative control shRNA (shNC) were purchased from Gene-Pharma Co (Shanghai, China). The target sequences for shMIIP and shNC were: shMIIP, 5′-AGGAGTTTCGGGAAACCAACA-3′. shNC, 5′-GTTCTCCGAACGTGTCACGT-3′. pRK5-HA-Ubiquitin-WT (#17608), pcDNA3.1-beta-3(#27289) vectors were purchased from Addgene (Cambridge, MA, USA). pGEX-4T-1 and pcDNA3.0 plasmids were kindly provided by Prof. Chengchao Shou (Beijing Institute for Cancer Research), and pGEX-4T-1-MIIP, pGEX-4T-1-MIIP^ΔRGD^, pcDNA3.0-MIIP and pcDNA3.0-MIIP^ΔRGD^ recombinant plasmids were then constructed. Human recombinant MIIP protein was purchased from OriGene (Cat#: TP300779, Rockville, MD, USA).

### Antibodies

Rabbit polyclonal anti-human MIIP antibody (HPA044948) was purchased from Sigma-Aldrich (St Louis, USA). The ITGB3 (#13166), ITGAV (#4711), β-catenin (#8480), FAK (#13009), p-FAK(Tyr 576/577, #3281), p-FAK(Tyr 397, #8556), AKT(#4691), p-AKT(Ser 473, #9271), E-cadherin (#3195), N-cadherin (#13116), Vimentin (#5741), Claudin-1(#13255), Myc-tag (#2278, #2276 S), Ubiquitin (#3933), and the HRP-conjugated goat anti-rabbit antibodies were obtained from Cell Signaling Technology (Beverly, USA). The VEGFA antibody (ab46154) was purchased from Abcam (Cambridge, UK). The HA antibody (#11867423001) was from Roche (Basel, Switzerland). The HRP-conjugated β-actin (HRP-66009) and GAPDH (HRP-60004) antibodies were obtained from Proteintech (Chicago, USA). The HRP-conjugated goat anti-mouse IgG were purchased from ZSGB-BIO (Beijing, China) respectively.

### Cell culture and establishment of stably transfected cells

BT-549 and MDA-MB-231 cells were bought from Cell Bank of the Chinese Academy of Sciences (Shanghai, China). All cell lines were verified using short tandem repeat (STR) profiling method at Cell Bank of the Chinese Academy of Sciences, and tested for mycoplasma contamination routinely every 6 months. The cells were maintained in RPMI 1640 medium containing 10% fetal calf serum at 37 °C in a CO_2_ incubator. For establishing stable transfectants with MIIP, MIIP^ΔRGD^ overexpression, or knockdown of MIIP, MDA-MB-231 cells or BT-549 cells were transfected with pcDNA3.0-MIIP, pcDNA3.0-MIIP^ΔRGD^ or shRNA targeting MIIP respectively using Lipofectamine 2000 (Invitrogen), and stable cell clones were selected with G418 (0.7 mg/ml). The selected cell clones were maintained in culture medium containing 0.3 mg/ml of G418.

### Western blot analysis

Cells were lysed in RIPA lysis buffer (50 mM Tris–HCl, pH 7.5, 150 mM NaCl, 1% NP-40, 1 mM DTT, 1 mM phenylmethyl sulfonyl fluoride, 10 mM NaF, 1 mM Na_3_VO_4_, 1× protease cocktail). BCA Protein Assay kit (KeyGen Biotech Co, Ltd., Nanjing, China) was used to determine the protein concentrations. Equimolar amounts of protein was separated using 12% sodium dodecyl sulfate-polyacrylamide gel electrophoresis (SDS-PAGE), and then transferred to polyvinylidene fluoride (PVDF) membranes (Millipore, USA) for immunoblotting using primary antibodies followed by HRP-conjugated goat anti-rabbit or anti-mouse IgG (1:5000).The expression levels of target proteins was normalized using GAPDH or β–actin (ACTB) as internal reference.

### GST pulldown assay

Expression of pGEX-4T-1 empty vector, recombinant MIIP- or MIIP^ΔRGD^-expressing vectors in BL21 host *E.coli* were induced by 0.2 mM IPTG at 21 °C for 16 h. GST, GST-MIIP and GST- MIIP^ΔRGD^ were purified using GST agarose beads, followed by an overnight incubation with cell lysates of MDA-MB-231 cells in a rolling incubator at 4 °C. The next day, after three times washing with PBS. SDS loading buffer were added into the beads and boiled for 5 mins for subsequent western blot analysis using antibodies against ITGB3 and ITGAV.

### Immunoprecipitation

Total proteins of the cells were lysed using NP40 lysis buffer (Beyotime, Shanghai), and quantified by BCA protein assay. Primary antibodies against c-Myc tag (#2276 S) or β-catenin were incubated with 25 μl of Protein G magnetic beads (Invitrogen) at 4 °C for 1 h. Cell lysates with same amount of proteins were then added into the antibody-beads complex and incubated at 4 °C overnight. The second day, after three times wash with NP40 lysis buffer, SDS loading buffer were added into the beads and boiled for 5 mins and subjected to western blot analysis.

### Quantitative real-time PCR

Total RNA of cells was isolated using TRIZOL Reagent (Invitrogen) according to the manufacturer’s instructions, and reversed to complement DNA by a Reverse Transcription System (Promega, Madison, WI, USA). Quantitative real-time PCR (qRT-PCR) reaction system was composed of 50 ng of cDNA template, 10 pmol of forward and reverse primers, and 10 μl of 2×SYBR®Premix Ex Taq II (TaKaRa Bio Inc., Otsu, Japan) to make a final volume of 20 μl. The reaction was performed using the ABI 7500 Fast Real-Time PCR system (Applied Biosystems, Carlsbad, CA). Primer sequences used in the experiment includes MIIP-forward: 5′-GACTGGATTGCAGGGTCTCT-3′, MIIP-reverse: 5′-TGGCTGCAGATACACTCCTC-3′; GAPDH-forward: 5′-GGACTCATGACCACAGTCCA-3′, and GAPDH-reverse: 5′-CCAGTAGAGGCAGGGATGAT-3′. Each sample was assayed in triplicate and the cycle threshold (Ct) values were normalized to GAPDH. Fold expression level of MIIP was calculated using the 2^−ΔΔCt^ formula.

### In silico analysis of clinical significance of MIIP in breast cancer

Illumina HiSeq RNA sequencing (RNA-Seq) data of TCGA Breast Cancer (BRCA) cohort, which has been normalized and processed by log2 (norm_count+1), were downloaded from UCSC Xena database. Then, the expression matrix of 1094 tumor samples from the RNA-Seq data were sorted out by TCGA barcodes and the average MIIP expression value was set as the threshold to split MIIP-high-expression (MIIP^high^) and MIIP-low-expression (MIIP^low^) cohorts. Differentially expressed genes between MIIP^high^ and MIIP^low^ groups, defined as |log2FoldChange | >0 and *P* value<0.05, were subjected to GO enrichment analysis using the ‘clusterProfiler’ package (v4.0.5) [44] in R software (version 4.1.2). The ‘genefu’ R package (v2.24.2) [45] based on PAM50 classifier [46] were used for breast cancer subtyping to identify the expression level of MIIP in different subtypes. MIIP gene alteration was analyzed using the Cancer Genome Atlas online database which was queried by cBioPortal (https://www.cbioportal.org/). The database of UALCAN (http://ualcan.path.uab.edu) was used for survival analysis of MIIP in breast cancer with different subtypes.

### MTS assay

Triplicates of 1000 cells in 100 μl medium were plated into each well of the 96-well plates. At 24 h, 48 h, 72 h, and 96 h, cell proliferation was assessed by CellTiter 96® Aqueous One Solution Cell Proliferation Assay (Promega, Madison, WI, USA) according to the protocol provided by the manufacturer.

### Colony formation assay

Triplicates of 1000 cells were plated into the each well of 6-well culture plate, incubated for 7–10 days until cell colonies formed. Then the wells were washed with PBS, fixed for 30 min with methanol, and stained with 0.5% crystal violet solution. The number of colonies (>50 cells/colony) in each well were then counted under an inverted microscope.

### Cell cycle analysis

Cells were digested with trypsin, harvested and fixed in 75% ethanol overnight at −20 °C. After being treated with 0.1 mg/mL RNase A for 30 min, cells were then stained with 50 μg/mL PI and analyzed by a FACS Calibur system (Becton Dickenson). The results were analyzed using ModFit LT 3.0 (Verity Software House Inc., Topsham, ME).

### Human angiogenesis array

Human angiogenesis array was performed according to the manufacture’s protocol (#ARY007, R&D systems, USA). Briefly, MDA-MB-231 cells with or without MIIP overexpression were collected and lysed using the lysis buffer provided in the Kit. Cell lysates were then incubated overnight with the pre-blocked array membrane at 4 °C. The second day, the array membranes were washed using wash buffer to remove unbound proteins followed by incubation with a cocktail of biotinylated detection antibodies for 2 h at room temperature. Then after a thorough wash of the membrane, streptavidin-HRP and chemiluminescent detection reagents were applied to the membrane, a chemiluminescent signal was produced at each captured spot corresponding to the amount of phosphorylated protein bound. The pixel density of each spot was analyzed and quantified using Image Lab software (Bio-Rad).

### Collection and preparation of conditioned medium (CM)

MDA-MB-231 or BT-549 cells with different MIIP expression levels were cultured in RPMI 1640 medium plus 10% FBS until confluence. Then the medium were replaced with 10 ml of serum-free RPMI 1640 medium. After 48 hr incubation, the medium were collected and sequentially centrifuged at 4 °C, 2000 rpm for 20 min and 7000 rpm for 20 min to fully remove cellular debris. The supernatants were then subjected to ultrafiltration on the 10-kDa cut-off membrane (ultrafree-15/0.5 10 K, Amicon Ultra Millipore) for concentrating the conditioned medium (CM). Concentration of the CM was measure using BCA protein assay.

### In vitro tube formation assay

Human umbilical vein endothelial cells (HUVECs) were starved for 24 h before the assay. Ibidi u-slide angiogenesis plates (IBIDI, USA) were precoated with 10 μl Matrigel (10 mg/ml; BD Biosciences, USA) and incubated at 37 °C for at least 1 h. 50 μl of HUVECs (2 × 10^5^ cells/ml) in DMEM medium plus 4 μg of conditioned medium (CM) from MDA-MB-231 or BT-549 cells with different MIIP expression levels were seeded onto the well of u-slide plate. The plates were then incubated at 37 °C for 6 h, capillary-like tube formation was observed and photographed under inverted microscope, and analyzed using the ImageJ software.

### Chick embryo chorioallantoic membrane assay

Fertilized chicken eggs were purchased from a local hatchery and placed in an incubator and maintained at 37 °C, 50% humidity until day 6 of embryo development. The eggshells were then cleansed with prewarmed 75% ethanol, and a small square of approximately 1 cm^2^ was drilled in the location of air sac. Afterwards, the chicken chorioallantoic membrane (CAM) was separated from the shell membrane, then the shell of the egg was carefully removed, and a created window was sealed with a sterile cellophane tape. The eggs were placed back into the incubator. The next day, 4 μg CM of breast cancer cells were placed onto the CAM using sterilized filter paper, and the eggs were incubated for an additional 24 h or 48 h. The neovascularization of the CAM were observed and photographed.

### Rat aortic ring assay

The thoracic aorta of Sprague-Dawley rats (6 weeks old) was dissected and cut into 1–2 mm long and set into the wells of 48-well plates which were precoated with 100 μl of Matrigel. Then, additional 100 μl of Matrigel solution were added into the well to form a sandwich structure. The plates were incubated at 37 °C, 5% CO_2_ for 30 mins for solidification. Afterwards, 300 μl ECM medium containing 4 μg CM of breast cancer cells were added into the plates. The plates were placed in the incubator at 37 °C and 5% CO_2_. The medium were changed every two days. The sprouting vessels were observed and photographed under inverted microscope.

### Xenograft mouse model

MDA-MB-231 or BT549 cells with altered MIIP expression and control cells (5 × 10^6^ cells/0.1–0.2 ml PBS per mouse) were injected subcutaneously into 4-week-old SPF grade female BALB/c nude mice respectively. The tumor volume was monitored every seven days by measuring the longest diameter (L) and the shortest diameter (W) with a caliper and calculated using the formula: L × W^2 ^× 0.5. The maximum diameter in any dimension of the tumor masses measured is less than 1.5 cm. The mice were euthanized by cervical dislocation under carbon dioxide anesthesia (CO_2_, 70% chamber volume replaced per minute) 28 days after injection. The death of mice was confirmed by observing respiratory and cardiac arrest, and the lack of pupil dilation and nerve reflex. For metastasis study, the MDA-MB-231 cells (2 × 10^6^ cells in 0.2 ml PBS) were intravenously injected into 4-week-old female nude mice. 5 weeks later, the mice were euthanized, and the lungs were removed and fixed in 4% paraformaldehyde for hematoxylin and eosin (HE) staining.

All animal experiments were conducted following the institutional ethical guidelines on animal care and approved by the Ethics Committee of Ningxia Medical University (reference number: 2014-007).

### Immunohistochemistry staining

A breast cancer tissue array formed from tissues of consenting donors was purchased from Outdo Biotech (Shanghai, China). The tissue array or the formalin‑fixed and paraffin‑embedded xenograft tumor tissue sections were heated at 65 °C for 1 h, then deparaffinized in xylene and rehydrated in a series of graded ethanol. Endogenous peroxidase activity was blocked with 3% hydrogen peroxide at room temperature for 10 min to block, and EDTA buffer (pH 8.0) was then used for antigen retrieval. The sections were blocked with 10% goat serum (Beyotime Biotechnology, Shanghai, China) for 10 min at 37 °C, followed by incubation with primary antibodies at 4 °C overnight. The next day, after washing 3 times with PBS, the sections were incubated with HRP-conjugated secondary antibody for 1 h at room temperature. Specific detection was developed with 3′3-diaminobenzidine (DAB). The immunoreactivity score was evaluated blindly by two independent pathologists.

### Statistical analysis

Statistical analysis was performed by GraphPad prism version 6.0 (GraphPad, San Diego, CA, USA). Data are presented as mean ± standard deviation (SD) of three biologically independent experiments or samples. Two-tailed, Student’s *t*-test for independent samples was used to assess difference between two groups. Difference was considered to be statistically significant when *P* value was less than 0.05.

## Supplementary information

Supplementary figures

Supplementary WB data

Author contribution form

Checklist

## Data Availability

The data generated in this study are available within the article and its supplementary data files. Additional data related to this paper are available upon request from the corresponding author.
