# Supplementary material for: MIIP functions as a novel ligand for ITGB3 to inhibit angiogenesis and tumorigenesis of triple-negative breast cancer
Source: Cell Death Dis. 2022 Sep 21;13(9):810. doi: 10.1038/s41419-022-05255-0 (PMC9492696; doi:10.1038/s41419-022-05255-0)
Supplement: Supplementary file 1 — Supplementary figures [file 41419_2022_5255_MOESM1_ESM.docx]

**
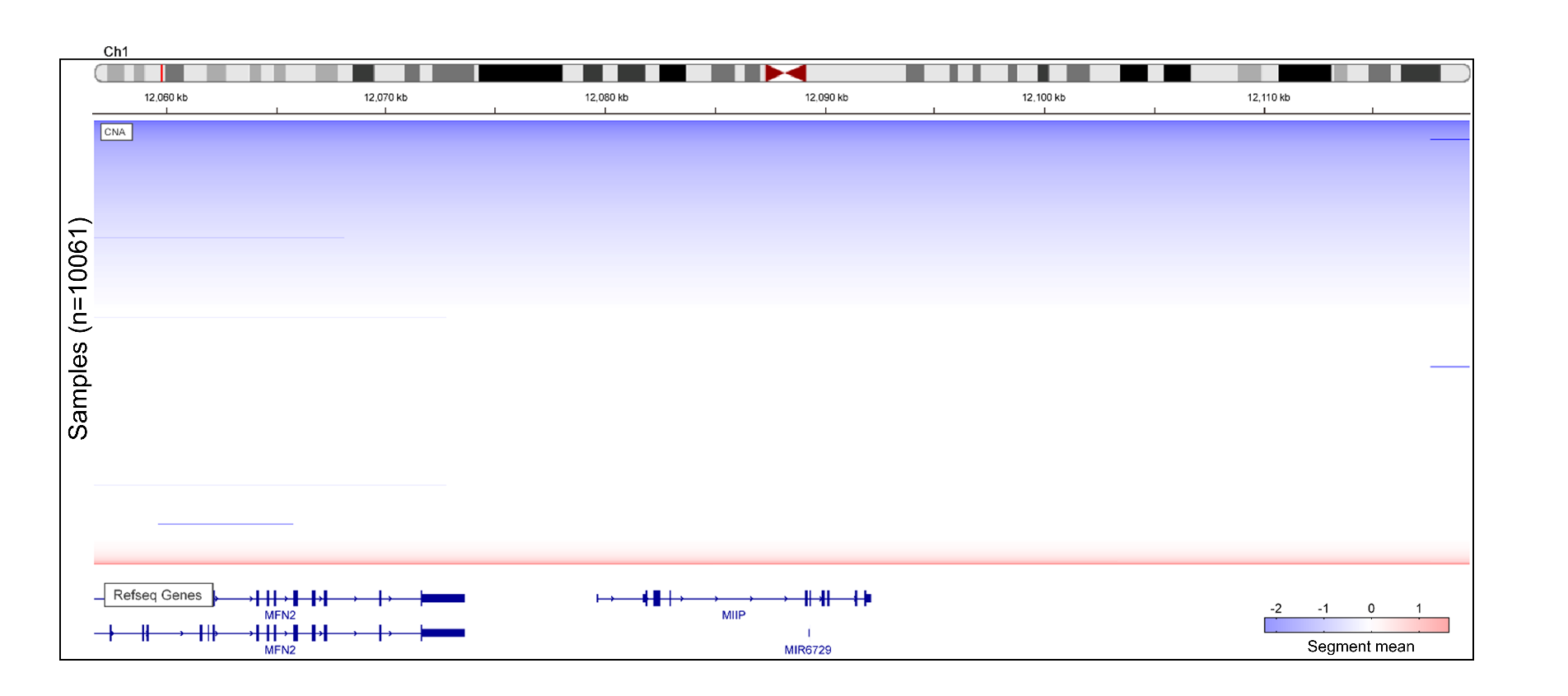
Supplementary Figures**

**Supplementary Figure 1.** DNA copy number of *MIIP* at 1p36 was plotted using the TCGA dataset in cBioPortal. Samples are ranked according to *MIIP* copy number, expressed as the mean log ratio of the segment encompassing *MIIP*. Genes present in that genomic regions are shown (bottom panel).


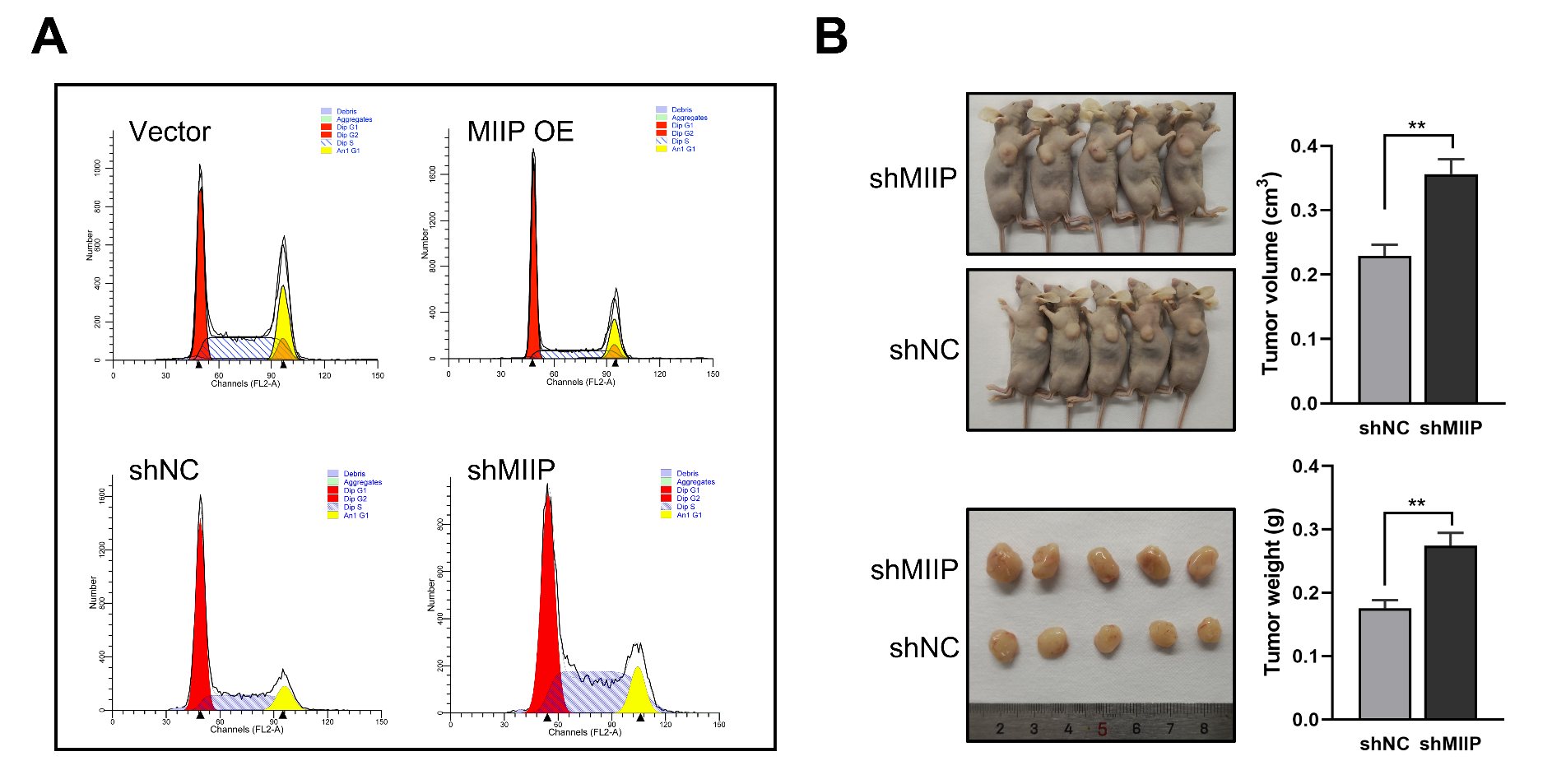


**Supplementary Figure 2. (A)** Representative cell cycle images of MDA-MB-231 and BT-549 cells with altered MIIP expression. **(B)** BT549 cells with or without MIIP knockdown were injected subcutaneously into nude mice. Images of the tumor-bearing mice and tumor masses are shown. Tumor volume at day 28 and tumor weight were compared between shNC and shMIIP groups.

**
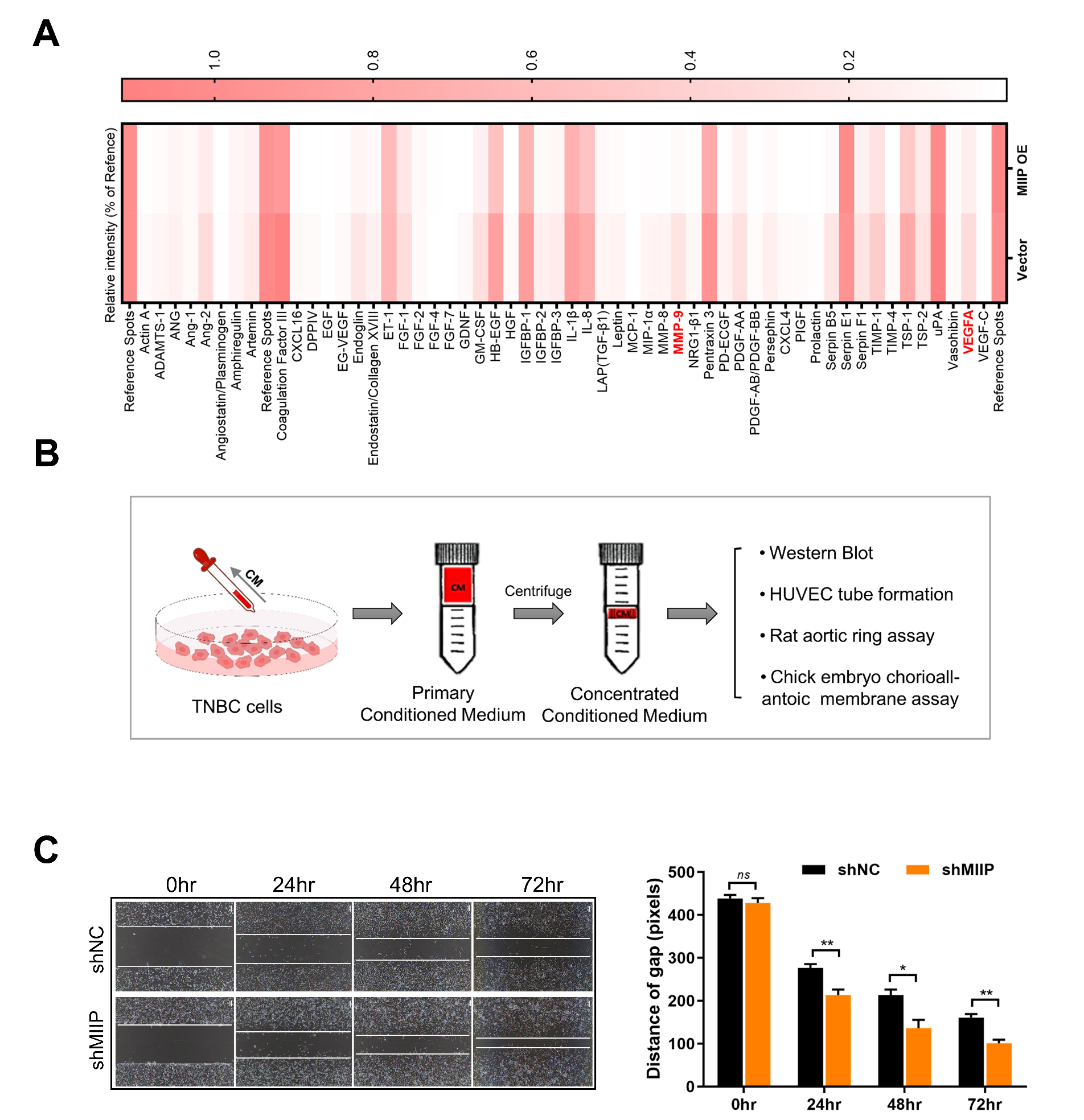
Supplementary Figure 3.** **(A)** Heat map showing the quantification of Human angiogenesis array in Fig. 3C. **(B)** A schematic showing the preparation of conditioned medium (CM) for angiogenesis analysis. **(C)** Wound healing assay was performed to evaluate the migration ability of BT549 cells with or without knockdown of MIIP.

**
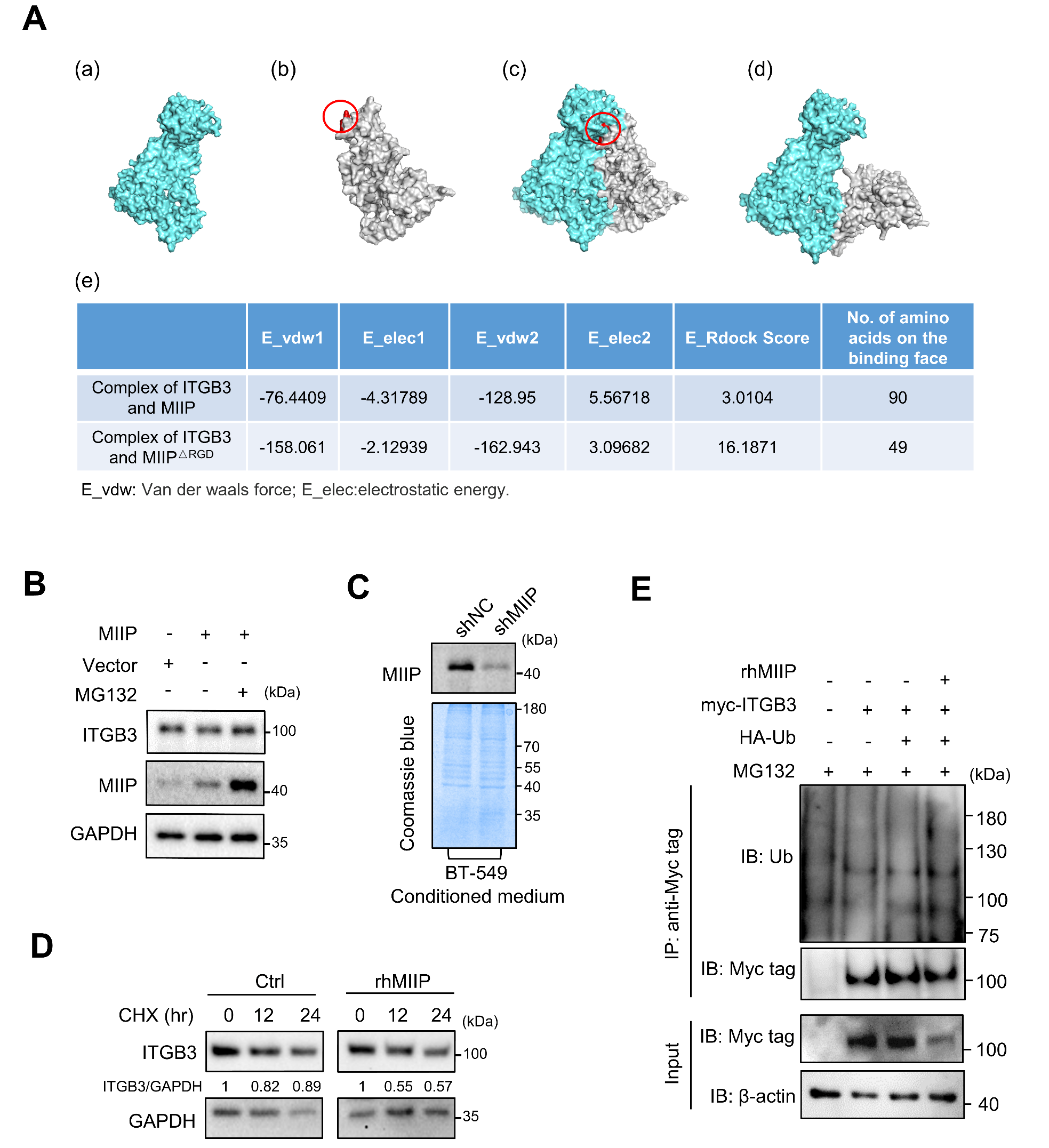
**

**Supplementary Figure 4. MIIP interacts with ITGB3 and promotes its degradation. (A)** Computational analysis of the interaction between MIIP and ITGB3. (a) Structure of ITGB3 (PDB ID: 6BXJ); (b) the tertiary structure of MIIP constructed using the trRosetta server (https://yanglab.nankai.edu.cn/trRosetta/), with RGD motif highlighted in red circle; (c) Generated models for interaction of ITGB3 and MIIP or (d) MIIP^△RGD^ using ZDock program of Discovery studio (Version 4.5); (e) Binding affinity of ITGB3-MIIP complex and ITGB3-MIIP^△RGD^ complex was compared based on interaction energy, E-Rdock score, and number of amino acids on the binding face. **(B)** ITGB3 protein levels were measured in MDA-MB-231 cells with indicated treatment. **(C)** Protein levels of MIIP in the conditioned medium of BT-549 cells stably transfected with indicated constructs were determined by western blot. Coomassie blue staining of the gel was applied to serve as loading control. **(D)** Effect of rhMIIP on the stability of ITGB3 was investigated in MDA-MB-231 cells with 100 μM treatment of CHX for the indicated time points. **(E)** Effect of rhMIIP on the ubiquitination of ITGB3 was investigated in MDA-MB-231 cells with 10 μM treatment of MG132.
