# Supplementary material for: MIIP functions as a novel ligand for ITGB3 to inhibit angiogenesis and tumorigenesis of triple-negative breast cancer
Source: Cell Death Dis. 2022 Sep 21;13(9):810. doi: 10.1038/s41419-022-05255-0 (PMC9492696; doi:10.1038/s41419-022-05255-0)

Fig. 2A

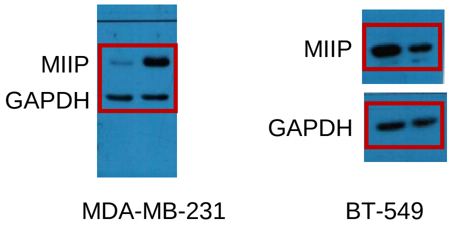

Fig. 2F

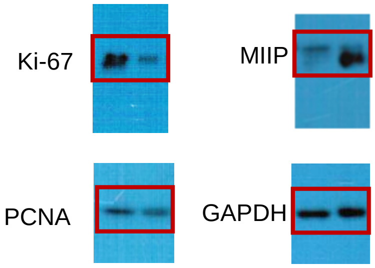

Fig. 3D

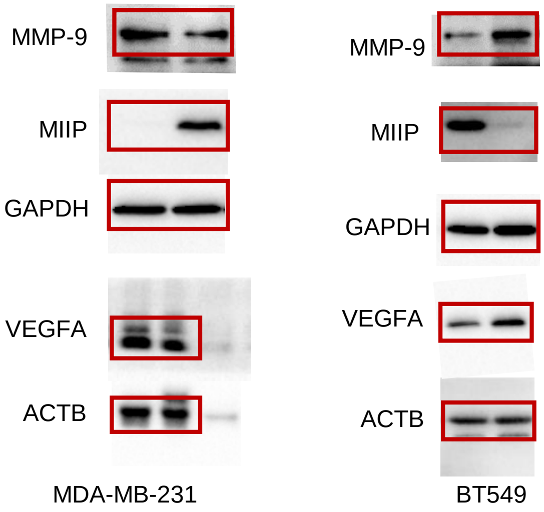

Fig. 3E

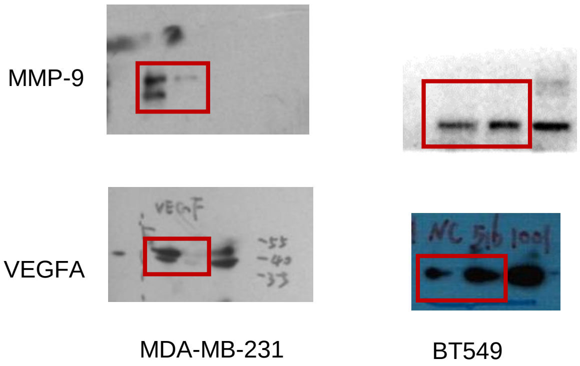

Fig. 3K

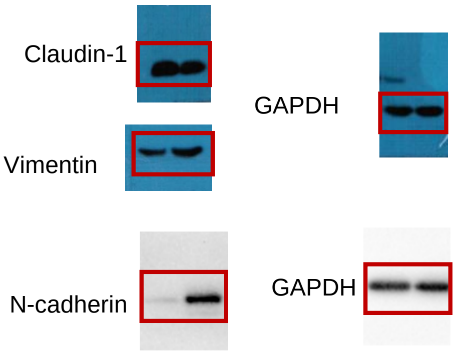

Fig. 4B

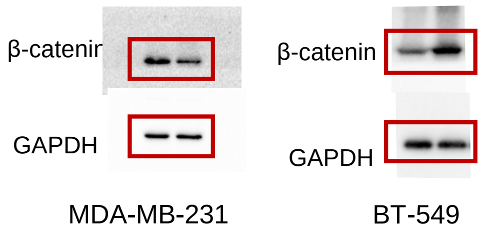

Fig. 4C

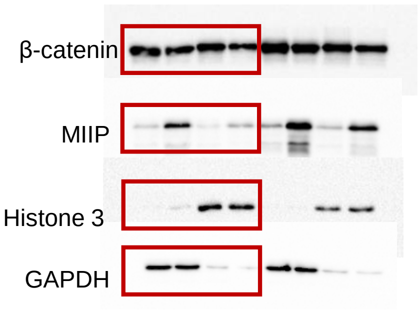

Fig. 4F

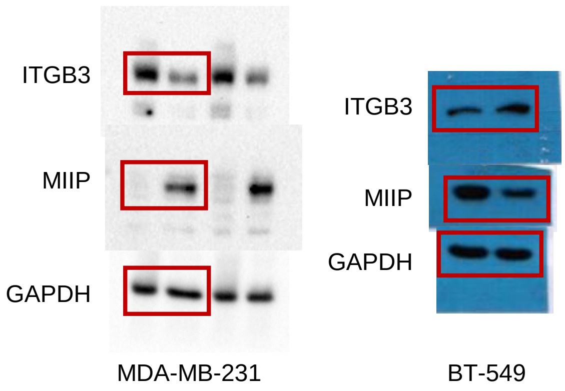

Fig. 4G

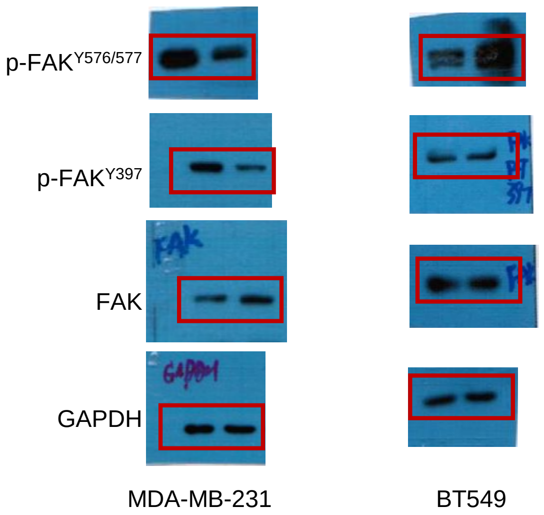

Fig. 4H

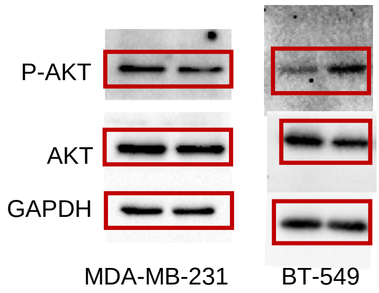

Fig. 4I

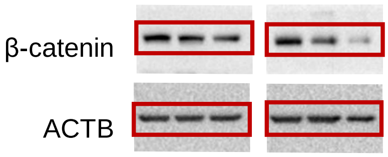

Fig. 4J

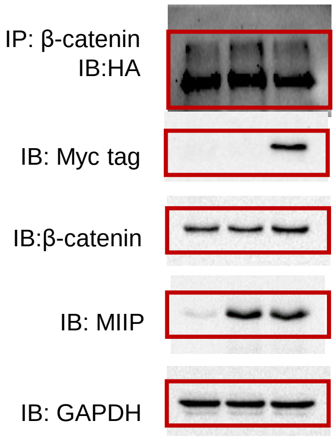

Fig. 4K

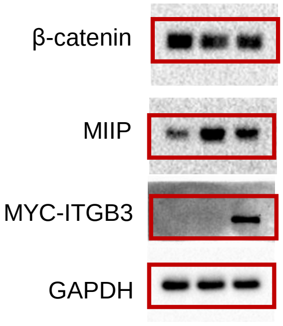

Fig. 5B

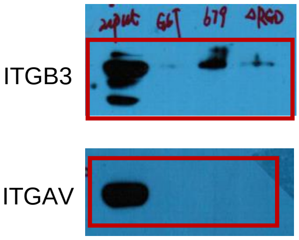

Fig. 5C

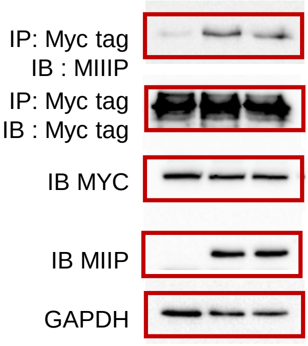

Fig. 5D

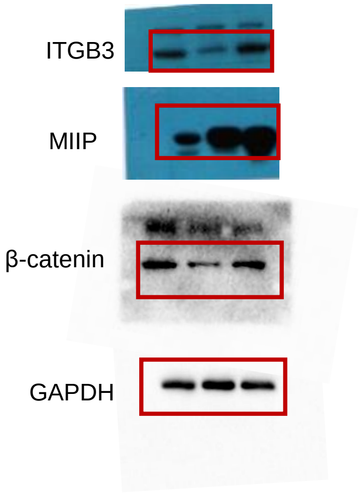

Fig. 6G

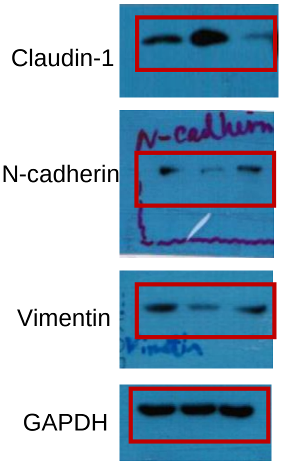

Fig. 5E

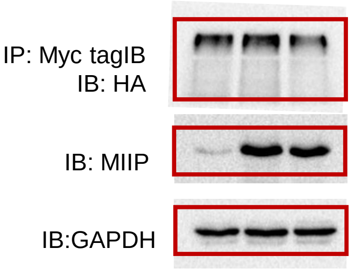

Fig. 5F

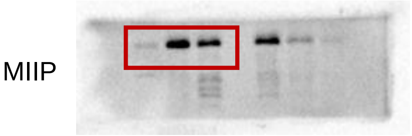

Fig. 5G

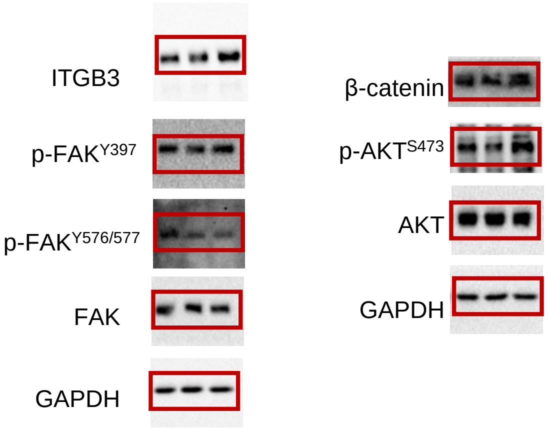

Fig. 5H

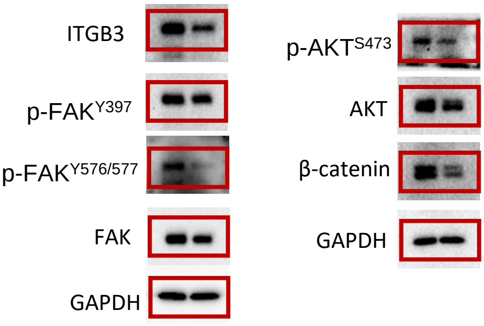

Fig. S4B

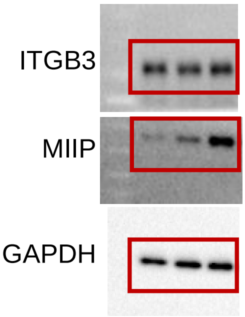

Fig. S4C

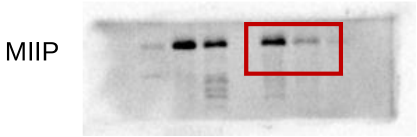

Fig. S4D

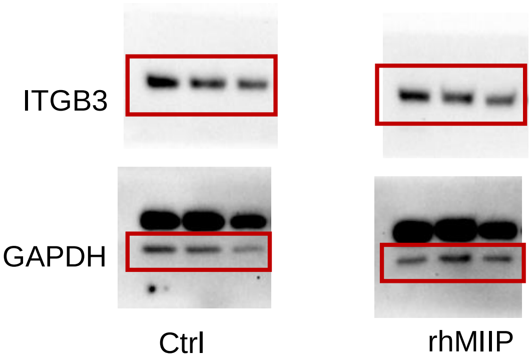

Fig. S4E

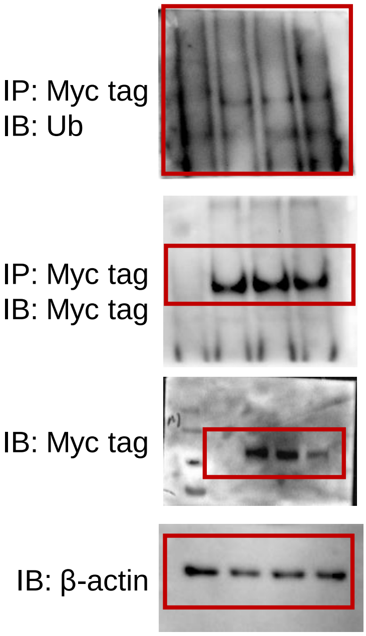

Supplement: Supplementary file 2 — Supplementary WB data [file 41419_2022_5255_MOESM2_ESM.pdf]
