# Supplementary material for: MIIP functions as a novel ligand for ITGB3 to inhibit angiogenesis and tumorigenesis of triple-negative breast cancer
Source: Cell Death Dis. 2022 Sep 21;13(9):810. doi: 10.1038/s41419-022-05255-0 (PMC9492696; doi:10.1038/s41419-022-05255-0)
Supplement: Supplementary file 3 — Author contribution form [file 41419_2022_5255_MOESM3_ESM.pdf]

DECLARATION OF CONTRIBUTIONS TO ARTICLE

ADMC

Manuscript Number:	Journal Name:	
CDD-22-0998	*Cell Death & Differentiation*	(the 'Journal')

Proposed Title of the Contribution:

MIIP functions as a novel ligand for ITGB3 to inhibit angiogenesis and tumorigenesis of triple-negative breast cancer (the 'Contribution)

Author(s):

Yujing Gao , Yujie Fang, Yongli Huang, Rui Ma, Xixi Chen, Fang Wang, Xiuying Pei, Yuanqi Gao, Xuehua Chen, Xinrui Liu, Jingxuan Shan, Pu Li (the 'Authors')

For all *CDD* articles, each person named as an author in the published version must be able to show he or she has contributed substantially to the article.

Authorship credit should be based on 1) substantial contributions to conception and design, acquisition of data, or analysis and interpretation of data; 2) drafting the article or revising it critically for important intellectual content; and 3) final approval of the version to be published. Authors should meet conditions 1, 2 and 3.

Any person who cannot be shown to have made a substantial contribution to the article cannot be listed as an author in the final version. The name of any person who is deemed to have made a minor contribution can, however, appear in the Acknowledgments section of the article.

Please complete the table below to indicate the contributions of all named authors to the manuscript.

Author Full Name:	Specification of Contribution to the Manuscript:
Yujing Gao	Performed study concept and design. Performed the research. Analyzed the data. Wrote and revised the paper.
Yujie Fang	Performed the research. Analyzed the data. Wrote the original draft.
Yongli Huang	Performed the research. Analyzed the data.
Rui Ma	Performed the research. Analyzed the data.
Xixi Chen	Data collection and analysis.
Fang Wang	Data collection and analysis.
Xiuying Pei	Provided technical and material support.
Yuanqi Gao	Data collection and analysis.
Xuehua Chen	Performed the research.
Xinrui Liu	Provided technical and material support.
Jingxuan Shan	Performed study concept and design. Revised the paper.
Pu Li	Performed study concept and design, and experiment. Revised the paper.

Please complete the table below to indicate the contributions of all named authors to the figures.

Figure 1:

In Figure 1, Xixi Chen, Yujing Gao and Fang Wang collected the data through TCGA database and generated the data. Pu Li performed experiments. Fang Wang prepared all panel and assembled the figure.

Figure 2:

In Figure 2, Yujing Gao, Yujie Fang, Yongli Huang, Xuehua Chen, and Pu Li performed the experiments and analyzed the data. Yujing Gao prepared all panel and assembled the figure.

Figure 3:

In Figure 3, Xixi Chen performed the bioinformatic analysis. Xiuying Pei designed the method. Yujing Gao, Rui Ma, Pu Li and Yujie Fang performed the experiments and analyzed the data. Yujing Gao prepared all panel and assembled the figure.

Figure 4:

In Figure 4, Xixi Chen performed the correlation analysis. Jingxuan Shan and Pu Li designed the method. Yujing Gao, Yujie Fang and Yongli Huang performed the experiment and analyzed the data. Yujing Gao prepared all panel and assembled the figure.

Figure 5:

In Figure 5, Yuanqi Gao performed protein structure analysis. Pu Li and Jingxuan Shan designed the method. Yujing Gao, Yujie Fang, Xinrui Liu performed the experiment and analyzed the data. Yujing Gao prepared all panel and assembled the figure.

Figure 6:

In Figure 6, Yujing Gao, Yujie Fang and Rui Ma performed the experiment and analyzed the data. Yujing Gao prepared all panel and assembled the figure.

Signed for and on behalf of the Author(s):	Print Name:	Date:
Yujing Gao	Yujing Gao	05/15/2022
